# Recommencement of football competition with spectators during the active phase of the COVID-19 pandemic in a Middle Eastern country

**DOI:** 10.1186/s13102-022-00504-3

**Published:** 2022-06-20

**Authors:** Naushad Ahmad Khan, AbdulWahab Abubaker Al Musleh, Sameer Abdurahiman, Mohammad Asim, Ayman El-Menyar, Hassan Al-Thani

**Affiliations:** 1 Department of Surgery, Trauma and Vascular Clinical Research, Hamad General Hospital, P.O. Box 3050, Doha, Qatar; 2 Clinical Information Systems (CIS), Hamad Medical Corporation and Medical Affairs, Supreme Committee for Delivery and Legacy, Doha, Qatar; 3 Department of Clinical Medicine, Weill Cornell Medical College, Doha, Qatar; 4 Department of Surgery, Trauma and Vascular Surgery, Hamad General Hospital, Doha, Qatar

**Keywords:** COVID-19, Risk-mitigation, Public health, RT-PCR, Soccer, Rapid antigen, SARS-CoV-2, Sports management

## Abstract

**Background:**

With the global spread of COVID-19 infection caused by the severe acute respiratory syndrome coronavirus-2 virus (SARS-CoV-2), all the national and international sports events were ceased early in 2020. The sport activities have been reinstated since then, albeit without spectators. However, several governments have established a variety of risk-mitigation measures to gradually reintroduce the spectators to stadiums.

**Objectives:**

We aimed to evaluate the implementation of a strict health protocol to ensure the resumption of professional football with spectators and to access its effectiveness in limiting the spread of COVID-19 infections within the community.

**Methods:**

This was a retrospective, observational study involving football players, match officials, local organizing committee members, working in close coordination, and over 16,000 spectators in the state of Qatar. We examined data from the Amir Cup final (December 18th, 2020), which was played under a strict protocol that included extensive reverse transcription-Polymerase chain reaction (RT-PCR) testing for players and match officials, as well as the utility of COVID-19 rapid antigen and antibody testings as screening tools for spectators to ensure their safe return to the stadiums. In addition, we reviewed the guidelines and protocols that were put in place to organize Qatar's Amir Cup Football Final, which drew over 16,000 spectators in the stadium.

**Results:**

A total of 16,171 spectators undertook rapid antigen and antibody tests for the Amir cup final (from December16-December18, 2020). Fifteen Spectators (n = 15) returned with a positive result for COVID-19 infection during the final event (positivity rate = 0.12%). All players underwent RT-PCR testing 48 h before the match. None of the players tested positive for COVID-19 infections. 1311 individuals reported having symptoms related to COVID-19 post final of Amir Cup. These spectators were tested for COVID-19 RT-PCR with an overall positivity rate (positive/reactive) to be 0.42% (69/16171).

**Conclusion:**

This report shows a meagre incidence rate of COVID-19 infections during and post-Amir Cup football final. Based on the low infectivity rate reported during and post the Amir Cup, we propose that supervised and controlled resumption of football matches with spectators can be carried out safely following a strict testing and tracing protocol. Similar infection control policies can be replicated with a higher number of spectators.

## Background

In March 2020, the World Health Organization (WHO) declared COVID-19, an infection caused by the severe acute respiratory syndrome coronavirus-2 (SARS-CoV-2) virus, as a pandemic [1]. Following the outbreak of COVID-19, by April 2020, health authorities and governments in several countries declared confinement measures to mitigate the infection spread, which resulted in the suspension of all significant professional sports training and elite competition [2, 3].

Football, an immensely popular sport played and watched by billions of people worldwide, was also affected as other sports events involving mass gatherings were suspended at some point in almost every part of the world [4, 5]. There is an increasing debate about the professional football resumption during the COVID-19 pandemic era. These international professional football tournaments provide a mass spectacle for the public [6] while having significant health and socioeconomic consequences for the host nation [7], including an increased risk of infectious disease transmission [3, 8]. As a result, pandemics such as COVID-19 have raised the stakes for evaluating the effects of holding large-scale sporting events. Due to the global spread of the COVID-19 epidemic, Qatar's professional football leagues and all local sport activities were suspended from March until mid May 2020. Preventive approaches have become the mainstay of addressing individual-level risk control as more information regarding viral transmission pathways emerges and becomes more apparent with each passing day. However, when dealing with sports involving large crowds and professional players, these precautions might become impractical and difficult to implement, making social distancing quite challenging to follow [9, 10]. Hence, a strategy for a safe return to sporting events involving mass gatherings is required, as well as a defined degree of phased re-opening based on a structured spectator screening procedure. This would allow sporting activities to be held as they were before COVID-19, i.e. in a safe setting, with the intention of avoiding COVID-19 infection after the event [11, 12].

Despite Safety and health concerns, football leagues are slowly fostering up by many countries across the globe amidst the ongoing COVID-19 pandemic [1, 13, 14]. Qatar, a country in the Arabian Gulf, has played an instrumental role in building confidence amongst players and fans alike by hosting a couple of football tournaments. In May 2020, the Qatar government decided to create a task force composed of sports physicians, scientists, and health care professionals under one umbrella to allow the resumption of the football league and implement a return-to-competition protocol. In fact, Qatar was one of the first countries to host spectators during national sport activities following the declaration of COVID-19 as a pandemic [11, 15]. The Qatar Football Association (QFA), in conjugation with the Ministry of Public Health (MoPH), adopted preventative steps to help organize the Amir Cup Football Final 2020, which drew over 16,000 fans. This marked the phased resumption of football with spectators in Qatar during the COVID-19 pandemic and was indeed a watershed moment in organizing sport events , as it may serve as a model to other sporting bodies.

Currently, there is a gap in our knowledge regarding the impact of football matches with spectator attendance on the possible spread of COVID-19 infections. Evidence in the literature is scanty on the impact of fans gathering at sporting events more generally on the incidence of COVID-19 at the local level [16]. This study evaluated and described the gradual return to the competition using a strict protocol, involving the clinical and safety measures and operational plan undertaken in hosting the Amir Cup football final played with spectators during the active phase of COVID-19 in Qatar. Additionally, this article is a reply to a prior call of action [11] for the countries and organizations involved in conducting such events to provide the scientific community with the details of public health policies undertaken, protocol implemented and to publish real data on the post-event infection rate in the local settings. We believe that these data can provide valuable information regarding the controlled resumption of sporting events with spectators within the milieu of the active COVID-19 pandemic and preparation for the upcoming FIFA world cup 2022 in Qatar.

## Methods

A retrospective observational study was conducted. The study involved football players, match officials, local organizing committee (LOC) members, working in close coordination, and over 16,000 spectators from the Amir Cup final 2020 held in the state of Qatar. The QFA (Qatar Football Association) was responsible for Organizing the Amir Cup Event. We examined data from the Amir Cup final (December 18th, 2020), which was played under a strict protocol that included extensive SARS-CoV-2 reverse transcription-polymerase chain reaction (RT-PCR) testing for players and match officials, as well as the utility of COVID-19 rapid antigen and antibody testings as tools for screening spectators to ensure their safe return to the stadiums. In addition, we reviewed the guidelines and protocols that were put in place to organize Qatar's Amir cup football final, which drew over 16,000 fans in the stadium. This study was granted ethical approval from the Medical Research Centre (MRC) and institutional review board (IRB) of Hamad Medical Corporation (HMC), Doha, Qatar (IRB#MRC-01-21-431) that waived the requirement of informed consent. A waiver of consent was granted for this retrospective analysis as there was no direct contact with subjects, and data were obtained anonymously. It was not appropriate or possible to involve patients or the public in our research's design, conduct, reporting, or dissemination plans. This study follows the Strengthening the Reporting of Observational Studies in Epidemiology (STROBE) checklist.

We collected players, spectators, RT-PCR, Antigen and Antibody testing from the MOPH in Qatar. All Statistical and descriptive analyses of Amir cup final event 2020 and subsequent follow-up data about the COVID-19 infections post-event were performed using the Microsoft Excel version 2017 (Microsoft Office, Redmond, Washington, USA) and GraphPad Prism 9.0 (La Jolla, CA, USA).

### COVID-19 testing protocol for Players and match officials for Amir cup final

The current gold standard for detecting the presence of COVID-19 infection is RT-PCR testing [17, 18]. The test is susceptible and specific to SARS-CoV-2 viral RNA [19]. All PCR analyses were carried out at Communicable Disease Centre (CDC) Laboratory at HMC in Qatar, following standardized protocols. Nasopharyngeal swabs were collected. Reverse transcription-Polymerase chain reaction (RT-PCR) testing was performed on aliquots of Universal Transport Medium (UTM). Aliquots were extracted on the Qiagen (QIA) symphony platform (QIAGEN, USA) and tested with real-time reverse-transcription PCR (RT-qPCR) using the TaqPath COVID-19 Combo Kit (Thermo Fisher Scientific, USA) on an ABI 7500 FAST (Thermo Fisher, USA); using a custom protocol [20] on a Hamilton Microlab STAR (Hamilton, USA) and tested using the AccuPower SARS-CoV-2 Real-Time RT-PCR Kit (Bioneer, Korea) on an ABI 7500 FAST; or loaded directly to a Roche cobas® 6800 system and assayed with the cobas® SARS-CoV-2 Test (Roche, Switzerland). Interpretation of the results was performed as per the manufacturer's instruction based on the respective cycle threshold (cT) of the gene target amplified. Results were reported based on the cT value either positive (cT < 30), reactive (cT ≥ 30 and < 40), negative (> 40) and inconclusive. The average time of validation and availability of results from the time sample was taken for PCR testing was approximately eight hours. A team of trained and experienced nurses took the samples from all the players, match officials and spectators (Only post-event) in the presence of a representative from the concerned team or designee. PCR testing was performed 1–2 days before the match.

### Rapid Antigen testing and screening for spectators

Rapid antigen tests are a reliable screening method for COVID-19 in nasopharyngeal swabs [21]. The SARS-CoV-2 rapid antigen test is a reliable, chromatographic immunoassay for the qualitative detection of specific antigens of SARS-CoV-2 present in the human nasopharynx (SARS-CoV-2 Rapid Antigen Test Kit, Roche Diagnostics International AG, Rotkreuz, Switzerland). The test provides specificity of 99.2% and sensitivity of 95.5% (cT value ≤ 30) with a testing time of 15–30 min. In the current study, the sampling for diagnosing COVID-19 was mandatory and done through rapid antigen testing (RAT) among all the spectators before each match. Test results were analyzed and interpreted as per the manufacturer's instructions. A negative result indicated no coronavirus antigen in the specimen. Spectators with negative SARS-CoV-2 antigen were not required to undergo further PCR testing.

### SARS-CoV-2 Antibody testing and screening for spectators

SARS-CoV-2 specific immunoglobulin (IgG and IgM)) Antibodies were measured in serum samples using an electrochemiluminescence immunoassay (Roche Diagnostics, Rotkreuz, Switzerland) to identify seroconversion as a measure of contact with SARS-CoV-2 among spectators. Test results were interpreted as per the manufacturer's instructions, with a cut-off value ≤ 1 as negative and ≥ 1 as positive. Spectators with positive SARS-CoV-2 antibodies were not required to undergo further PCR testing.

### Return-to-stadium protocol and management for spectators for the Amir Cup final

**Fig. 1:**
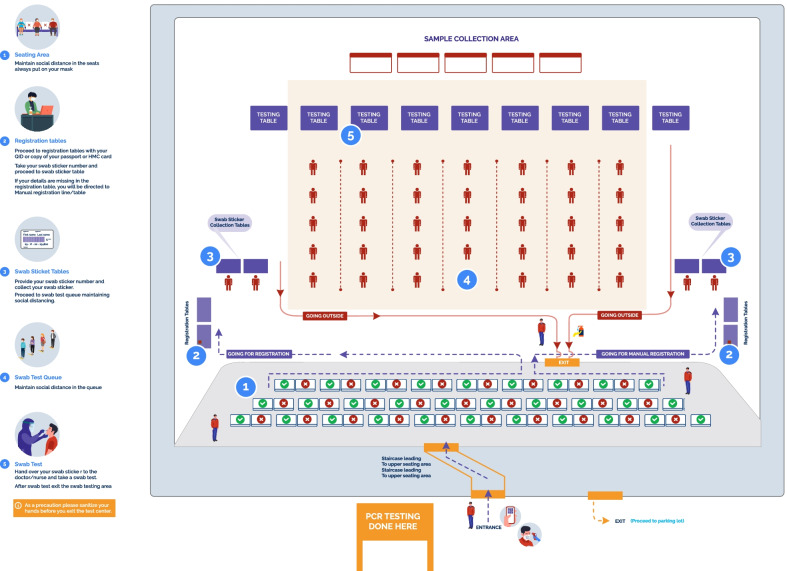
Illustration of the antigen testing for spectators’ setup at sport clubs

The largest stakeholder group at any match is the spectators, especially those at the stadium. The AFC (East) championship league was remarkable as it marked the partial return of spectators. The ticket allocations were limited to 50% of the overall capacity of the venue/stadiums in Qatar. Crowd management poses the most significant challenge during sporting events. During the Amir Cup final, special arrangements were made to mitigate the transmission of COVID-19 during the event. Before being admitted inside the stadium, social distance, temperature screening, and mask-wearing were required (non-pharmaceutical interventions, or NPIs). All spectators intended to witness the final had to show either a negative COVID-19 test or a positive COVID-19 antibody test. Temporary testing centres for SARS-CoV-2 rapid antigen test were put up at the football clubs and in an adjacent conference centre in Doha 72 h before the event. All antigen testing for spectators was performed at Qatar National Convention Centre (QNCC), Al-Sadd, Al-Arabi, Al-Rufaa, Cricket stadium and Al-Rayyan (only local organizing committee (LOC)) maintaining proper social distancing (Fig. 1).

**Fig. 2:**
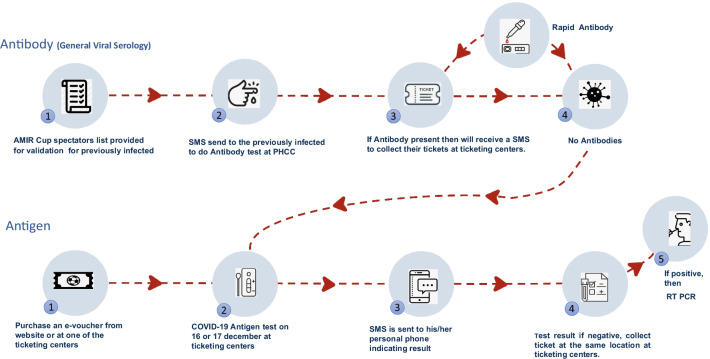
Spectators pathway for Antigen and Antibody testing with implementation of organizational precautions to minimize the risk of transmission of COVID-19 infections during the Amir Cup Final 2020, Qatar

The ticketing centres and website set up for spectators to purchase tickets contained detailed written instructions on obtaining the tickets. Spectators were needed to purchase an online voucher, which they were to bring to the testing locations. At the testing facilities, all spectators were subjected to RATs and had to wait 15–30 min for the results sent to their registered mobile phones via SMS. Those who received a negative result were told to present the message and voucher to the ticketing hall facility, where they were awarded their match tickets. The tickets bore the Spectator's Qatar identity number and name, making them non-transferable. Spectators who had previously been infected with COVID-19 were asked to have an antibody test (blood test) performed at the Primary health care corporation (PHCC) (November 24th to December 5th) and later (December 13th to December 18th) in the same location. If antibody was present, they received SMS to collect their tickets at the ticketing centres, and if antibodies were absent, they had to undergo a rapid antigen test. If any spectator tested positive, they were promptly isolated and moved to the nearest COVID-19 health clinic to repeat the COVID-19 test using the RT-PCR, after which they would follow the Ministry of Public Health (MOPH's) isolation protocols and instructions in Qatar (Fig. 2).

Additionally, standard precautionary methods were followed at the stadium. It was done by implementing (1) Social distancing measures by reducing the overall numbers of spectators up to 50% of stadium capacity (2) Having a green status (COVID negative) in the geo-localization tracking app (3) Initial health screening procedures such as temperature and symptom (e.g., cough, shortness of breath, fever, chills, sore throat, headache, etc.), (4) Mandatory wearing of a mask at all times, (5) Fans seated on alternate seats (spacing between the two spectators was considered at least 1.5 m in front, behind, and diagonally to avoid crowding). (6) All high touch surfaces inside the stadiums were disinfected daily, and multiple mobile handwash stations and automated hand sanitiser dispensers were added to designated areas of stadiums. Overall, a comprehensive multi-layered protocol was strictly implemented to mitigate any possibility of transmission of COVID-19 infection during the Amir cup final competition with spectators. In Fig. 2, an illustration of the spectator's approach to attending the match is depicted.

## Results

### COVID-19 antigen and antibody testing for spectators

**Fig. 3:**
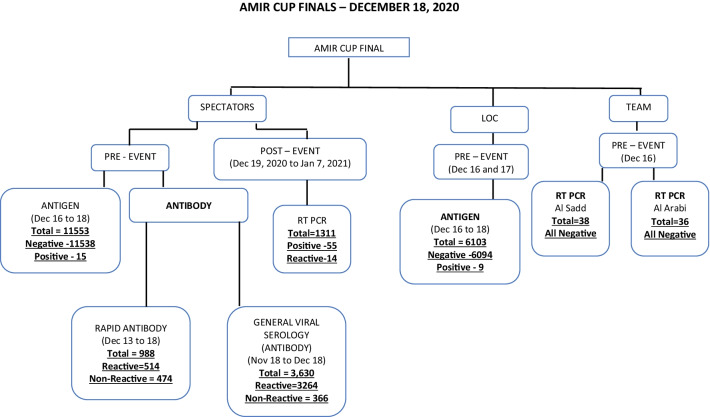
Schematic depiction and overall summary of the screening tests i.e. RT-PCR, 220 Rapid Antigen Test, Rapid Antibody test and General viral serology test employed for 221 the players match officials and spectators during pre and post Amir Cup Final, 2020, Qatar

A comprehensive safety net of expert planning, vigorous testing, and medical protocols were put in place to ensure the health protection of all stakeholders, including players, match officials, and spectators. A mass antigen and antibody testing protocol were implemented for spectators. Sampling was conducted for diagnosing COVID-19 through the rapid antigen testing measured in the nasopharyngeal swab. A total of 11,533 rapid antigen tests were done for the Amir cup final (From December 16-December 18, 2020). Fifteen spectators (n = 15) returned with a positive result for COVID-19 infection before the final event (positivity rate = 0.12%). Spectators who had previously been infected with COVID-19 were asked to have an antibody test (blood test) performed at PHCC (November 24 to December 5). Before the final event (Dec 13–18, 2020), a total of 988 individuals were tested for rapid antibody testing, with 514 (52.02%) found to be reactive and 474 (47.9%) as non-reactive. Those who were non-reactive were subjected to undergo a rapid antigen test, and if their result came negative, they could attend the event (Fig. 3).

Two teams participated in the final match (Al-Sadd and Al-Arabi). Thirty-eight (38) players from Al-Sadd team and 36 individuals from the Al-Arabi team underwent RT-PCR testing 48 h before the match (16 December 2020). None of the players from both teams tested positive for COVID-19 infections. With respect to the local organizing committee staff, a total of 6103 individuals underwent RATs, 48–72 h prior to the event, of which 09 (0.14%) individuals returned with positive test before the final match.

### Monitoring of post-event infections among spectators

**Fig. 4:**
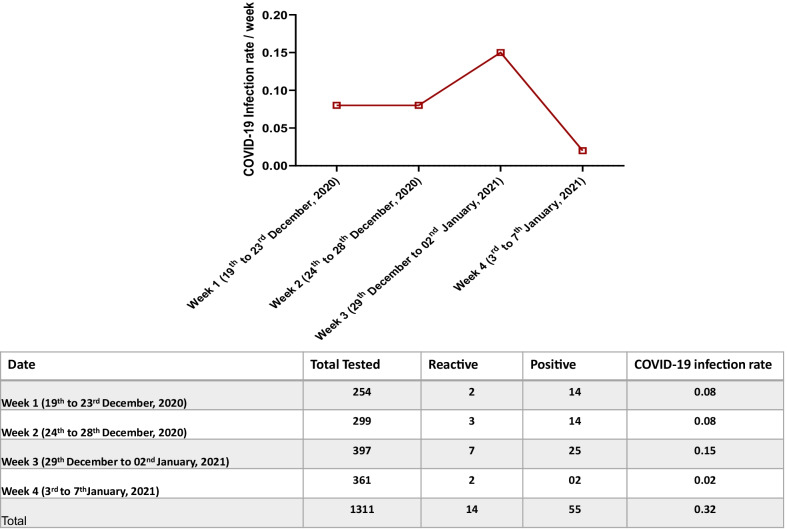
Summary of the RT-PCR test and overall positivity rate among the spectators who developed COVID-19 related symptoms post Amir Cup 2020

We analysed the health status of the spectators before and after the football event based on RAT/antibody testing/PCR-based surveillance. We followed up on the spectators' health status for four weeks post-event to determine the possible transmission of COVID-19 infection. It is understood that the length of time of transmission of COVID-19 to it presenting itself is around two weeks. It is further known that COVID-19 can remain asymptomatic in many individuals, and as such, if somebody gets infected with COVID-19 at a football match in mid-December, it is possible they may have passed the virus to others in early January whilst unaware, and the spread could be significantly more prominent as a result of the football match. The antigen test and monitoring findings were recorded using a geo-locating cell phone application (EHTERAZ) and an online booking system. Based upon this, a total of 1311 individuals reported having symptoms related to COVID-19 after the Amir Cup final match. These spectators were tested for COVID-19 RT-PCR with an overall positivity rate (positive/reactive) to be 0.32% (69/21,204) Fig. 4. We cannot conclude whether the spectators who came positive from the COVID-19 post final event were missed by the screening protocol or were infected from other sources after the event.

## Discussion

The experience of Qatar in hosting the Amir cup football final 2020 and the execution of preventive measures to reduce the spread of the COVID-19 infections among fans are described in this paper. Qatar was one of the first, Middle Eastern countries and worldwide to allow events of mass gathering including professional football with spectators during the active phase of COVID-19. It was a massive challenge considering that the WHO advisory warned the high risk of hosting an event involving mass gatherings during the active phase of the ongoing pandemic [22, 23]. During this unprecedented situation, the capability to safely resume the sporting events depends on implementing the safety measure for risk mitigation toward the spread and/or reinstatement of COVID-19, especially at times when there are high chances of newer waves of COVID-19 cases [24]. To our knowledge, the current study is one of the first to report the consequences for the spectators of a controlled resumption of competitive sport.

**Fig. 5:**
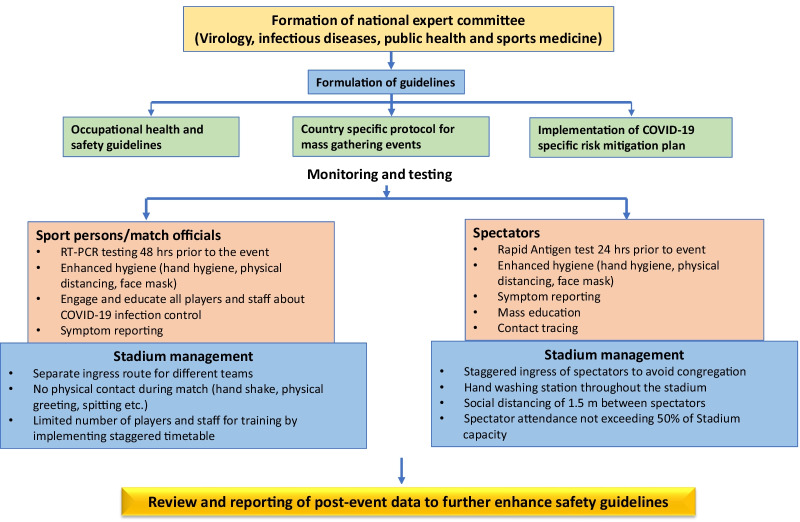
Algorithm depicting preventive strategies for conducting mass gathering events during COVID-19 pandemic

The Amir Cup event was concluded with 16,000 spectators in attendance in the same stadium and reported a meagre incidence rate of COVID-19 infections. This was the first time such an initiative was taken to welcome a huge number of spectators in a country that has not yet been deemed COVID-19-free. Qatar's robust and innovative long-term plan to contain the COVID-19 pandemic has offered free access to high-quality health care for locals and expats, highlighting the country's exceptional proactive leadership. Qatar had successfully executed its public health strategies and managed to control the outbreak of COVID-19 extremely, effectively and quickly [24, 25]. Figure 5 shows an algorithm depicting preventive strategies for conducting mass gathering events during the COVID-19 pandemic. At the start of the pandemic, Qatar was one of the countries with the highest infection rates per million population (the infection rate peaked at 1.27% on 27 May 2020 with about 35,634 active cases for a population of 2,807,805), but by 18 December 2020, it had dropped to about 0.07% (2090 positive active cases among the entire population).

Under such epidemiological conditions, our study found that resumption of professional football under strict adherence to the return to competition protocol and a gradual return of a small number of spectators to stadiums were not associated with spread of the COVID-19 infections.

Since the COVID-19 pandemic began in 2019, there have been few reports of high transmission of COVID-19 cases during athletic events other than soccer. Outdoor events with large crowds, such as professional football, can behave as super-spreaders of an airborne virus like COVID-19 [16, 26]. There is a lack of evidence about the impact the attendance of fans under a controlled environment can have on mitigating the super-spreader nature of mass sporting events during the COVID-19 pandemic [26]. Ahammer et al. [3] and Cardazzi et al. [27] determined the impact of sporting events as super spreaders. Ahammer et al. looked at mass indoor events in the COVID-19 pandemic and found that these events led to around 380 more COVID-19 cases and 16 more deaths per one million people in the country. During the Amir cup-2020 final event, none of the players was tested positive for COVID-19 infection. This is remarkable because stadiums present unprecedented challenges to COVID-19 mitigation strategies due to the involvement of many factors such as the sheer size of attendance (50%), seating proximity, high level of contact between athletes, and the spectators' intensity.

A study with similar intent looked at the impact of the virus's spread during April 2020 from the English football matches played in February and March 2020, before the first national lockdown. The evidence suggested that regardless of how full the stadiums were, the health outcomes following an English football match in March were consistent; for every 100 000 people in the same local area, there were 6 COVID-19 cases, 2 deaths, and 3 overall deaths [28]. Subsequently, the UK government postponed the planned reintroduction of spectators in September 2020, with resumption set to begin on October 1st, 2020. This decision was made in response to the greater COVID-19 restrictions in the UK, after various clubs hosted test events with approximately 2000 spectators [16]. However, due to the changes in the situation with COVID-19, the govt instructed sporting bodies that spectators would not be allowed into stadiums until at least March 2021.

In contrast to the scenario mentioned above, our experience showed that if a multi-layered protection strategy involving physical separation, disinfection, communication, and crowd control is strictly implemented, professional football with spectators in stadiums can be successfully re-booted in the face of the COVID-19 pandemic.

Our findings are consistent with those from the German Bundesliga professional football league. They reported a successful return of the game under a controlled environment with a diminished risk of viral transmission [13]. Indeed, Schumacher et al. observed that during the COVID-19 pandemic, the Qatar main football leagues (League 1 and 2) were restarted, with no indication of COVID-19 transmission from player to player during training or match play. However, both the abovementioned events were organized without spectators.

In our setting, matches were played with 50% attendance. Even though studies have shown antigen testing to be highly accurate in detecting positive cases, no factual inference can be drawn from our data regarding the danger of COVID-19 infections during match play involving spectators. Nonetheless, there is a higher possibility of false-negative outcomes. The latter is likely to raise worries about testing. Any positive antigen result will result in a PCR confirmation test, although a false negative might conceivably allow the undetected spread to occur. As a result, spot PCR validity testing on negative antigen samples are required as a precaution. When fans are present at sporting events, daily testing should be conducted as an additional layer of surveillance safety. However, we must acknowledge that even in countries where the surge of COVID-19 infections has initially been contained, the subsequent wave of new infections involving new strains has been reported [29, 30]. In such a scenario, the return to play protocol which ensures more robust infection mitigation measures assumes greater importance to create the safest possible environment for the return professional football with spectators.

### Strengths and limitations

One of the strengths of our study is that we analysed the health status of the spectators before and after the football event based on PCR based surveillance and followed up on the spectators' health status for four weeks post-event. We assume that spectators who tested positive for COVID-19 after the final event may have been missed by the screening protocol or were infected from other sources after the event. To mitigate this, we recommend performing rapid antigen tests within 24–48 h before sports events. A possibly positive spectator who got the virus after testing might infect other spectators after 2 or 3 days. So even if he has slipped through the net, the other spectators may still be relatively secure because the positive spectator is not infective yet [6, 31, 32]. Also, we recommend that the players and match officials to undergo rapid antigen test before the last training session prior to the game. Any suspected positive cases need to be validated by PCR. The rapid antigen test has the advantage of requiring fewer logistics, being less expensive, and providing better accuracy regarding the contamination risk before the game, i.e. 24 h instead of 48–72 h, when there is a chance of players contracting an infection following the test. We caveat our analysis by noting that the stadium access and egress routes can be adapted. Some of the opportunities for the spread of an airborne virus such as SARS-CoV-2 could be mitigated.

Lack of asymptomatic surveillance and voluntary reporting were the two important limitations of the study. Another, limitation is the relative delay in the time of publication as it came out almost one and half year post-event. We recognize that a timely publication can be critically important, especially in the fast moving field of research related to public health. However, we cannot account for the delay in publication which may be likely influenced by various factors beyond the control of the authors. Finally, this study took place prior to the start of extensive vaccination drive and the emergence of the SARS-Cov-2 Delta strain in Qatar.

Even though, the rate of COVID-19 positivity among spectators in our study was found to be very low, our results suggest extreme caution while returning to unrestricted spectator attendance at football matches. Nonetheless, the current article's conclusions should only be applied to outdoor sport settings, such as football games. The resumption and completion of the Amir Cup-2020 final acted as a template for the few other football tournaments involving international participants and spectators to be held successfully in Qatar. The game's safe and infection-free hosting marks an important milestone in Qatar's preparations for the FIFA World Cup, 2022. It provides an opportunity to test its operational plans further and ensure its readiness for football's showpiece event in 2022 amidst the world's uncertain epidemiological circumstances. Lastly, a recent study showed that the psychological support for sportsmen during the pandemic should highlight the coping strategies and sense of coherence; this issue was not addressed in our local experience, but it deserves further studies [33].

The current manuscript was written at the moment when much about the future of sport involving spectators remains uncertain, and seen from a specific set of perspectives and circumstances arising from the global COVID-19 pandemic. Twenty-one months later since the emergence of COVID-19, once more, chaos has descended upon the world of sports. Even some of the healthiest and most highly vaccinated communities are being ravaged by the emergence of the new COVID-19 variant Omicron [34]. A vaccination program has already been initiated in Qatar at the time of this manuscript writing and 86.9% of the Qatari population 12 years and above had already received two doses of vaccine, and 783,801 individuals have received booster dose vaccine [35]. The vaccination program remains the biggest hope to direct a series of pilot and research projects like the one we discuss in the current study [36, 37]. Both are expected to serve as a model for sports leagues to resume with spectators. It will ensure safety and provide a scheme for the public to psychologically get past the fear of being in large crowds after such an extended period of being social physically distanced and could act as a catalyst to win back the support of increasingly sceptical football fans. FIFA Football world cup is expected to be held in the winter from 21 November to 18 December 2022, and with Omicron surging around the world, sports once more must adapt. We have learned a lot about what types of public health interventions are effective in preventing the outbreak and based on our experiences; we can safely say that masking, distancing, and frequent testing really could thwart the new threat posed by the latest variant of the virus. Furthermore, researchers have started to propose public health and infection control policies to host a safe FIFA World Cup 2022 in Qatar [38, 39].

## Conclusions

A controlled reopening of professional football looks safe for spectators, players, and others engaged, as the chance of catching COVID-19 infection is low. However, this assumes that careful surveillance of probable spread is conducted, with all stakeholders involved acting responsibly and adhering to risk mitigation guidelines and recommendations. Even yet, professional sports leagues and related outdoor activities must continue to monitor public health statistics, particularly when new variants of the virus continue to spread and data on vaccination’s long-term effectiveness emerges. Additionally, this report calls again on the countries and organizations involved in conducting such events to provide the scientific community with the details of public health policies undertaken, protocol implemented as infection-risk mitigation measures and publishing real data on the post-event infection rate status of the individuals in the local setting.

## Data Availability

Not applicable.

## Abbreviations

COVID-19: Coronavirus infectious disease
PCR: Polymerase chain reaction
SARS-CoV-2: Severe acute respiratory syndrome coronavirus 2
